# Imaging evaluation of hallux valgus

**DOI:** 10.1590/0100-3984.2020.53.3e2

**Published:** 2020

**Authors:** Clarissa Canella

**Affiliations:** 1 Adjunct Professor of Radiology at the Universidade Federal Fluminense (UFF), Niterói, RJ, Radiologist specializing in musculoskeletal imaging at the Clínica de Diagnóstico por Imagem (CDPI) and the Clínica Alta Excelência Diagnóstica (ALTA), Rio de Janeiro, RJ, Brazil. Email: clacanella@yahoo.com.br.

Hallux valgus is a common foot deformity, affecting approximately 36% of adults over the age of 65, mainly occurring among women^(1,2)^. This condition is defined as medial deviation of the first metatarsal bone, lateral deviation of the hallux, and a prominent first metatarsal head. Hallux valgus can be caused by instability and insufficiency from the distal phalanx to the talonavicular joint, anywhere along the first ray^(1,3)^. The most important static stabilizers on the medial side of the hallux are the articular capsule, collateral ligaments, and medial sesamoid ligament. Insufficiency in any one of those structures leads to the development of the deformity and is considered an essential initial lesion. Hallux valgus is associated with disabling sequelae, including nerve entrapment, intermetatarsal bursitis, cartilage damage, and articular degeneration. The condition has a multifactorial etiology, often being associated with occupational risks and the use of inappropriate shoes, as well as with predisposing (variant) anatomy and genetic factors.

Radiography is considered the gold-standard imaging modality for the evaluation of hallux valgus. Radiographic measurements are essential parameters for assessing the severity of hallux valgus. The parameters most commonly used in clinical practice are the hallux valgus angle (HVA) and the intermetatarsal angle (IMA), because they have been shown to assess the severity of hallux valgus and to correlate best with the magnitude of deformity. The HVA is defined as the angle formed by two lines: one running from the midpoint of the first metatarsal head to the base and another running from the midpoint of the first proximal phalanx head to the base. The IMA is defined as the angle formed by a line running from the midpoint of the first metatarsal head to the base and a line running from the midpoint of the second metatarsal head to the base.

The evaluation of the musculoskeletal system by magnetic resonance imaging (MRI) has been the subject of a series of recent publications in the radiology literature of Brazil^(4-8)^. Although MRI is not considered the gold standard for the evaluation of hallux valgus, because MRI scans are not acquired during weight bearing, we agree with Helito et al.^(9)^ that MRI could be used for the diagnosis of this condition, given its high prevalence and the frequent unavailability of radiographic studies during weight bearing, as stated in their study published in the previous issue of **Radiologia Brasileira**. Although the angles have been widely described on radiographs, the MRI measurements of hallux valgus have not been so assessed. In their article, Helito et al.^(9)^ stated that the metatarsophalangeal angle (MPA) on MRI, with a cutoff point of 16.4º, can be used in order to diagnose hallux valgus with satisfactory accuracy. The MPA is defined as the angle formed by the intersection of the longitudinal axis of the first metatarsal bone diaphysis and the first proximal phalanx. The authors assessed the accuracy of MRI measurement of the MPA for the diagnosis of hallux valgus, using plain radiography during weight bearing as the gold standard. That information is important and clinically useful, because the authors established an objective MPA cutoff point to be incorporated into clinical practice for the diagnosis of hallux valgus, leading the radiologist to be active in reporting this condition. The authors also evaluated the interobserver agreement for MPA measurement on MRI, reporting that the level of that agreement was excellent.

The data presented by Helito et al.^(9)^ support the findings of Heineman et al.^(1)^ who demonstrated excellent interobserver agreement for the HVA and for the IMA on radiographs obtained during weight bearing and on MRI. Over the range of values that they tested, MRI was considered a viable imaging modality for diagnosing and assessing the severity of hallux valgus.
